# Multi-zone patterning enables hematocrit-independent precision metering of dried plasma for decentralized diagnostics

**DOI:** 10.1039/d5lc00844a

**Published:** 2025-11-17

**Authors:** Amanda Code, Giorgio Gianini Morbioli, Keith R. Baillargeon, Jack Soloway, Yanran Sun, Charles R. Mace

**Affiliations:** a Department of Chemistry, Tufts University Medford MA 02155 USA charles.mace@tufts.edu

## Abstract

There has been a notable increase in decentralized sample collection to facilitate diagnostic testing or wellness screening conducted by clinical laboratories. Plasma—not blood—is desired to access a majority of the diagnostic testing menu available to patients. Ideally, samples of plasma, which are dried after collection to facilitate stable specimen transport by mail, contain discrete and reproducible volumes that are independent of patient hematocrit and maintain the high purity expected from liquid plasma. We have developed a suite of plasma metering cards (PMCs) that can divide microsampled plasma from whole blood into multiple sample zones, enabling the metering of reproducible volumes across a wide range of hematocrits (30–55%). Sample zones can be used individually or aggregated together for assays that may require higher volumes. Critically, we show that a four-zone PMC outperforms a commercial plasma microsampling card (ADx 100 Serum Separator Card) in terms of three key performance criteria: total recovered sample volume (25.2 *vs.* 20.2 μL), sampling precision (CVs of 9.2% *vs.* 25.8%), and plasma quality (69% *vs.* 26% characterized as below mild hemolysis). These PMCs have the potential to support the rapid growth of decentralized clinical testing by empowering patients to self-collect high-quality dried plasma samples.

## Abstract 2

We developed a plasma metering card (PMC) that divides plasma from whole blood into multiple sample zones, enabling the metering of reproducible volumes across a wide range of hematocrits to support the rapid growth of decentralized clinical testing.

## Graphical abstract

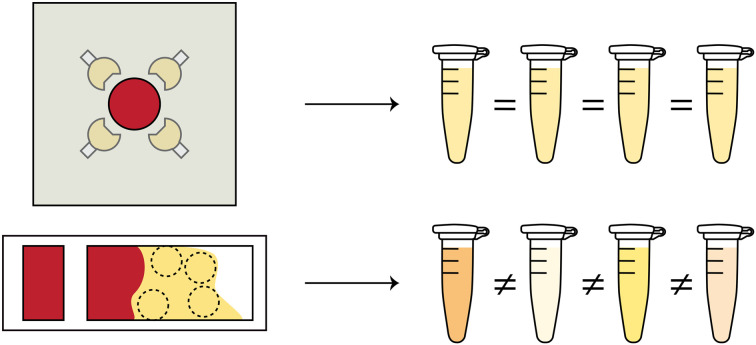

**Tribute to George Whitesides**

“*Who cares?*” This seemingly simple question caused a lot of anxiety during our group meetings, but the importance of its answer cannot be overstated. The problems we choose to dedicate our lives to should have meaning, and we should be able to communicate that significance clearly. I was fortunate to have the opportunity to practice this process across a range of projects—magnetic levitation, aqueous multiphase systems, and even instant flame suppression—before transitioning to attacking challenges in global health using paper-based microfluidics at Diagnostics For All, a nonprofit diagnostics company that George helped found. The combined opportunities and mentorship that George provided have enriched and shaped my career immensely. As I continue working and teaching at the intersection of technology and public health, I look forward to challenging the next generation of scientists by asking them, “*Who cares?*”

## Introduction

There is a growing push to decentralize blood collection to expand the scope of testing,^1^ support better population representation in clinical trials,^2^ and improve diagnostic opportunities in limited-resource settings.^3^ Despite the success of dried blood in supporting sample collection from a finger or heel stick,^4^ transportation of blood to laboratories under ambient conditions,^3^ and long-term specimen storage in biobanks,^5^ clinical laboratories often prefer plasma over blood as the sample matrix for testing soluble biomarkers.^6^ By removing cells, plasma is substantially depleted of common assay interferents (*e.g.*, hemoglobin) and analytes whose presence could bias a diagnosis (*e.g.*, potassium for cardiac health).^7^ In pharmacokinetic studies, cellular concentrations of the analyte can obscure the intended measurement, the fraction of the drug unbound in plasma, reducing vital information about bioavailability.^8^ Plasma is also frequently used for test panels,^9^ and individual analytes could benefit from unique samples with more specific eluents.^10^ Although numerous commercial options are available for microsampling blood and there is a high clinical demand for liquid plasma, very few microsampling cards exist for dried plasma.

Microsampling cards designed to collect blood cannot be utilized to collect plasma without first integrating some mechanism to remove blood cells from the liquid plasma before drying the sample. Traditionally, to generate plasma, a lab technician will process a vacutainer containing anticoagulated blood (*e.g.*, EDTA or heparin) by centrifugation to separate cells from plasma by density. In microsampling cards, plasma is generated passively. Cells are removed most commonly with size exclusion media,^11^ though alternative methods such as *in situ* agglutination, and material-induced clotting have also been used to generate plasma.^12,13^ These separation techniques can be integrated into existing microsampling technologies, allowing plasma to be processed and subsequently collected into a porous material. The separated plasma is then dried, which creates a static snapshot of a patient's health status and removes the need for cold chain transport to the receiving laboratory. Once at the laboratory, samples are removed from the card, usually *via* a punch, and then rehydrated in an appropriate eluent to recover analytes for analysis.

Besides simply separating the cells from blood, the dried plasma sample recovered from these cards must be comparable to a reference sample of liquid plasma by four main metrics: (i) the volume of plasma collected and eluted from the dried spot must be known accurately and collected with precision such that this volume can be used to calculate the original concentration of the analyte in the patient's blood. During sample preparation, the volume of eluent is known with accuracy, while the volume of plasma it contains is assumed, mainly from the surface area of the punch. This assumption is critical to the diagnostic value of the measurement yet can introduce additional error and reduce precision. Guidelines issued through the Clinical Laboratory Improvement Amendments (CLIA) serve to help contextualize card performance with real-world metrics by designating the minimum analytical performance required for a given analyte.^14^ (ii) The volume of plasma collected should be independent of biological variance across a population of users. That is, there should be no dependence on the collected volume of plasma from the patient's hematocrit, which is the fractional packed cell volume of blood. Hematocrit can vary widely (30–55%)^15^ from patient to patient and will dictate the total amount of available plasma in a given blood sample. To define that volume, microsampling cards cannot require a patient-dependent correction factor based on hematocrit because that would require an additional measurement and significantly complicate testing. (iii) Sample volumes should be positionally invariant such that laboratory operators do not introduce bias when recovering a sample. Fixed sample zones can streamline workflows and enable equivalent replicates for use in panels of assays or confirmatory testing. (iv) Dried plasma must contain little to no hemolysate (*i.e.*, have a low hemolysis index) to ensure that the release of intraerythrocytic analytes does not influence measurements.^7,16,17^ The primary contaminant released by hemolysis is hemoglobin, which is known to be a major interferent in colorimetric assays, due to its broad absorbance spectrum,^18^ and molecular assays, due to its inhibition of polymerases.^19^ It is therefore imperative that microsampling cards generate clean plasma. Lysis can occur either through the drying process that produces the dried plasma spot or due to aberrant interactions between cells and the card materials.^20^ Learning from techniques developed for liquid samples, such as aspirating a known volume of plasma using a pipette or liquid handling robot, regardless of patient hematocrit, and good phlebotomy practices to avoid hemolysis during collection and processing,^21^ it may be possible to improve the collection of dried plasma.

Solutions to generate dried plasma approach these requirements in unique ways, often through the choices of which materials are used or how they are arranged within the card. Though a wide range of solutions have been developed to remove blood cells for in-line assays,^22^ plasma generated for collection must not be diluted significantly or otherwise altered to remain usable to the widest range of downstream analyses as possible. Specifically, plasma separation membranes used for plasma collection mostly differ in their orientation, be that laterally,^23,24^ vertically,^25–29^ or through a combination of the two.^30^ Each spatial configuration has its advantages and disadvantages regarding sample quality. Cards that separate plasma by lateral flow may tend not to cause noticeable hemolysis by visual inspection, but the amount of hemolysate recovered is highly dependent on the position of the punch.^24^ Moreover, analytes are transported a substantial distance in these cards (*ca.* 3 cm or more), which can cause chromatographic effects leading to uneven sampling and poor agreement between replicate recovered samples.^31^ Better estimates of the volume contained in a punch can be made from measurements of chloride,^23,32^ but determining this correction factor adds time and complexity to a laboratory workflow. Cards that separate plasma by vertical flow limit uneven sampling but can suffer from high levels of hemolysis. Hemolysis in these cards is due to the position of the separation media directly above the sample zone, which can lead to transfer of hemoglobin directly into the sample zone as red blood cells (RBCs) lyse during the drying process.^26^ This class of cards can collect plasma in one large sample zone with limited metering (*e.g.*, Roche PSC)^28^ or in smaller sample zones that offer increased precision (*e.g.*, Telimmune Duo).^33^ In the case of the latter approach, removing the separation media before the blood entirely separates can increase the metering capabilities and decrease the risk of hemolysis, but creates a separate piece of biohazardous waste, requires accurate timing from the user, and results in low sample yields (*ca.* 3 μL).^27,29^ Separation media can be arranged in other configurations, which combine these lateral and vertical strategies for improved performance. Microsampling devices with separation media configured with a wedge-like design direct the plasma separated from blood into a channel by capillary action, which produces high-quality plasma without being affected by chromatographic effects seen in paper cards.^29^ Using a hybrid approach to plasma separation could therefore produce technologies that maintain the advantages from each configuration while minimizing its disadvantages.

Recently, we have shown that techniques developed to create paper-based microfluidic devices (*e.g.*, wax patterning and multilayer laminates) can be used to produce dried blood spot and dried plasma spot cards that offer metered sample collection with minimal dependence on the hematocrit.^25,26,34,35^ Our patterned dried plasma spot cards uniquely take advantage of both vertical and lateral flow within the card to first separate the plasma from whole blood (vertically) and then direct it away from the separation media (laterally) to protect against hemolysis. The short distance (*ca.* 4.5 mm) between where plasma is generated and ultimately collected also limits chromatographic effects. We designed the previous versions of our dried plasma spot cards predominantly to demonstrate our overall approach to collecting dried plasma^25^ or generate a single, high-volume sample zone to support the accurate measurement of HIV viral load in a clinical setting.^26^ While both cards demonstrated low hemolysis in collected plasma, the volumes collected in sample zones were either reported indirectly and at a single hematocrit^25^ or had a modest hematocrit dependence, particularly at higher hematocrits.^26^ Similar to how patterning can divide whole blood into multiple sample zones that enable the collection of hematocrit-independent volumes,^34^ we hypothesized that using a similar strategy could lead to increased sampling precision and reduced dependency on the hematocrit for dried plasma.

We designed a set of plasma metering cards (PMCs) that separated and collected plasma into a discrete array of replicate sample zones (one to four). We show that reducing the sample zone size ultimately leads to a PMC that produces precise volumes of plasma that are independent of the hematocrit: for samples with hematocrits spanning a range of 30–55%, three- and four-zone PMCs collected volumes with almost half the variation as one-zone PMCs (*e.g.*, 9.5% *vs.* 17.6% CV). Moreover, we show that the sample zones from multi-zone PMCs can be aggregated to recover larger total volumes of plasma that nearly equal one-zone PMCs while retaining high precision (28.7 μL and 17.6% CV for one zone *vs.* 28.0 μL and 6.0% CV for aggregated samples from three-zone PMCs). We compared the performance of our PMC to a commercial predicate, Advance Dx 100 Serum Separator Card (ADx 100), in terms of the reproducibility, separation efficiency, hematocrit-dependence, and overall quality of recovered plasma. The operational mechanism of the ADx 100 card differs from the PMC, but, as this card is widely used in direct-to-consumer test kits that rely on self-collection of plasma, it is the most appropriate commercial predicate to evaluate the performance of our cards. We observed that the reproducibility of the four-zone PMC was superior to that of ADx 100 cards and, unlike the ADx 100 card, the total available volume in the PMC was independent of the hematocrit. Critically, the quality of the collected plasma was also superior in the PMC compared to the ADx 100, whereby a substantially smaller number of samples recovered from PMCs were characterized by containing minor hemolysis compared to the ADx 100 cards (31% *vs.* 74%). PMCs have the potential to expand the clinical applications that are suitable for decentralized self-collection by increasing precision and allowing a flexible format for collecting replicate samples from a single fingerstick.

## Experimental

### Card design

**Fig. 1:**
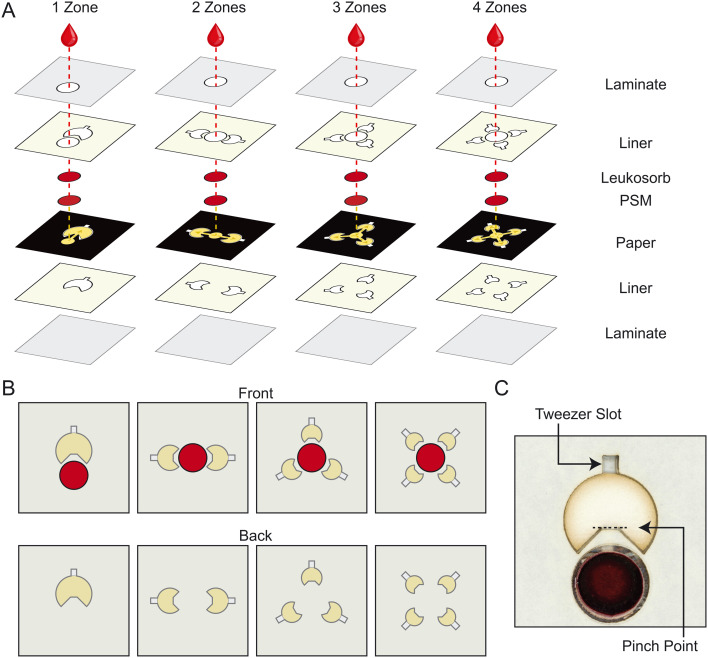
Design of plasma metering cards (PMCs) with patterns that divide collected plasma into multiple sample zones. (A) Exploded diagrams of card construction. The user adds 150 μL of blood to the inlet of the card which is created by a hole in the laminate film. The blood then flows through two layers of separation media (Leukosorb and a plasma separation membrane, PSM) to remove blood cells and allow the resulting plasma to flow into wax-patterned paper. The patterns split the plasma into pluckable sample zones. (B) A schematic view of the front and back of all four candidate PMCs. (C) Scanned image of a one-zone card after applying blood at a hematocrit of 40%. The arrow indicates the tweezer slot for plucking the sample zone out and the dashed line indicates the break point where the sample zone and channel will disconnect.

Leveraging previous versions of patterned dried plasma spot cards,^25,26^ we designed candidate PMCs that generated plasma through vertical separation before directing it laterally to the sample zone(s) composed of Whatman CF12 cardstock (Fig. 1A). These designs position plasma sample zones symmetrically around the separation media to limit any bias that could reduce sampling precision by utilizing the nature of paper to wick fluids isotropically (Fig. S1).^36^ We scaled the areas of sample zones such that the total sample collection area in each card was independent of the number of sample zones in the card (Fig. 1B) for a standard target input volume of blood (150 μL). Wax patterning, a standard method used to manufacture paper-based microfluidic devices, is known to introduce variability in the dimensions of features after the melting step,^37^ so we used a laser engraver to cut a physical border around each sample zone to define the area better and reduce variability attributed to the wax melting process. We designed sample zones in the shape of a major circular sector to help drive plasma into them by wicking.^38^ Additionally, we used laser cutting to incorporate a slot for tweezers (Fig. 1C) so that dried sample zones can be plucked directly at the pinch point, rather than punched from a card, to streamline the sample retrieval process.^26^

### Live subject statement

Samples of whole blood were supplied from healthy donors by Research Blood Components (Watertown, MA). All samples were tested for bloodborne pathogens and sample collection followed American Association of Blood Banks guidelines. This vendor is approved to provide collected blood for research purposes with IRB-approved consent and all research was approved by the Tufts University Institutional Biosafety Committee.

### Card operation and handling

We applied 150 μL of whole blood *via* a pipette onto the inlet of each candidate PMC (*n* = 1–4 zones) and the ADx 100 card in alignment with the manufacturer's instructions. We measured the volume and quality of collected plasma over six different hematocrits (30–55%; *n* = 8 cards per hematocrit per card type, Fig. S2), which we contrived from donor venous blood using previously reported methods (see the SI for details). After filling, we placed all cards into an environmental control cabinet (20 °C, 15% humidity) overnight to thoroughly dry. To process PMCs, we removed the sealing laminates and plucked individual sample zones from cards using tweezers. To process ADx 100 cards, we obtained four 6 mm punches from the membrane where the collected plasma looked cleanest by eye; unlike PMCs, it is not possible to recover plasma from a fixed position on the ADx 100 card because the membrane does not fill the same area across replicates or conditions (Fig. S2).

### Card characterization

We assembled PMCs manually and in small batches using previously described methods (see details in the SI). Consequently, we anticipated that we might observe minor rates of card failure due to the manual process of fabrication. To avoid propagating manufacturing errors into measurements and performance comparisons with the ADx 100 card, we first assessed the performance of PMCs using a visual quality check for proper filling. We excluded any cards that had incompletely filled plasma sample zones from further analysis and replaced them with a new card. We observed a failure rate of 11% across 216 total cards (Fig. S3). For those cards that were filled successfully, we used half of the sample pool (*n* = 4) to measure the volumes of dried plasma collected into each sample zone and the other half (*n* = 4) to measure the amount of hemolysate present in the recovered plasma. We determined the volume of plasma collected in sample zones by measuring chloride concentration in duplicate *via* a colorimetric assay. Chloride has been shown to normalize the plasma volume in dried plasma spot cards,^32^ so we used it as a proxy for plasma volume. While the volume of dried plasma can be determined gravimetrically, it lacks the sensitivity required for this experiment. In contrast, chloride measurements produced a linear relationship at the small volumes we would expect in the sample zones (Fig. S4). We pooled the plasma from three donors together to create a single calibration curve. Since we intended to compare the total sampling area of ADx 100 cards to the aggregated volumes acquired from sub-punches, we used image analysis to determine the total plasma volumes in these cards (Fig. S5). This analysis did not apply to our PMC since the available sampling area and aggregated volumes from all the sample zones are identical. Finally, we measured hemoglobin to characterize the quality of the dried plasma.^16^ We modified the procedure from a commercial kit (see details in the SI) to detect and quantify hemoglobin in the expected range for plasma containing no (<0.5 g L^−1^) or mild (0.5–3.0 g L^−1^) hemolysis (Fig. S6).

## Results and discussion

### Precision metering and distribution of collected plasma volumes in PMCs

**Fig. 2:**
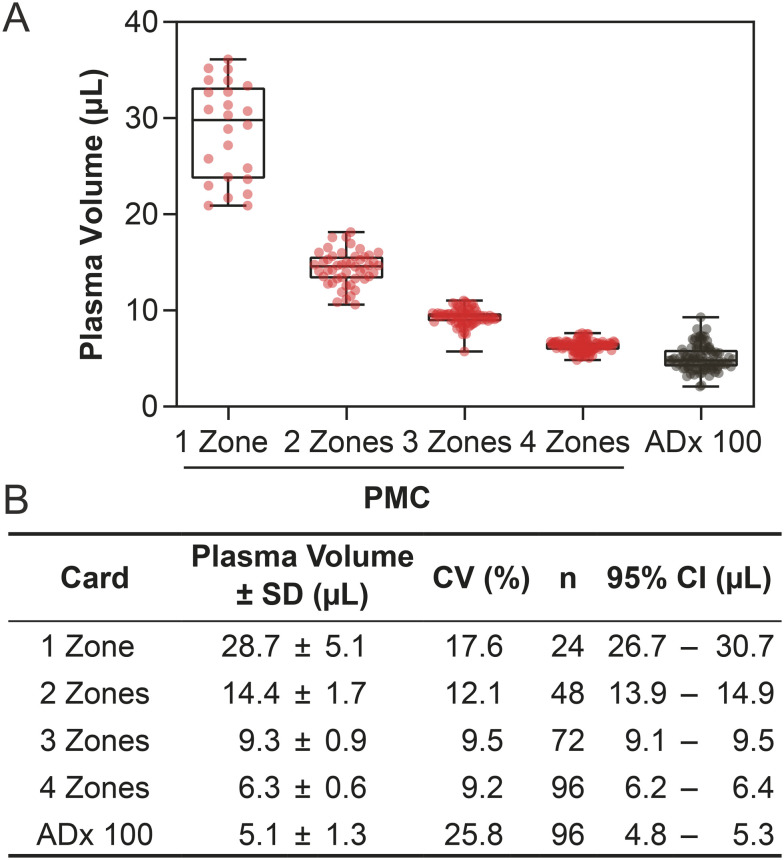
Measurements of plasma volumes extracted from individual sample zones from PMCs (various number of zones) and 6 mm punches from ADx 100 cards over the full range of hematocrits (30–55%). (A) Box plot of plasma volumes collected in different cards (PMC in red, ADx 100 in gray). The whiskers represent the minimum and maximum measured volumes. Plasma collected in PMCs was both higher in average volume and more reproducible than that in the commercial ADx 100 card. (B) Table of summary statistics from measurements of recovered dried plasma, including standard deviation (SD), coefficients of variation (CV), the number of replicate measurements for each card (*n*), and 95% confidence interval (CI). We tested 24 cards for each version of the PMC, so the number of samples increased as the number of zones in the card increased. Notably, the three- and four-zone samples had CVs below 10%.

The primary function of a PMC is to collect a reproducible volume of plasma that is independent of the position of the sample zone (for those cards with multiple sample zones) and the hematocrit of the patient. That is, PMCs should collect a known volume of plasma with high precision across a population.^39^ For routinely tested analytes, CLIA guidelines require accuracies ranging from 5–30% depending on the analyte.^14^ In order to be comparable with measurements taken from liquid samples aliquoted using a pipette, plasma separation cards must contribute negligibly to the overall variance of any given assay.^40^ We designed sample zones with defined areas such that they self-meter regardless of hematocrit. The results of metering experiments are shown in Fig. 2. One-zone PMCs generated plasma volumes of 28.7 ± 5.1 μL (CV 17.6%) across the full range of hematocrits tested (30–55%). Two-zone PMCs showed an increase in the precision of recovered plasma volume, yielding 14.4 ± 1.7 μL (CV 12.1%) per sample zone. This card had a confidence interval across all hematocrits that only varied by 1 μL. This trend continued for the three- and four-zone cards, which collected 9.3 ± 0.9 μL (CV 9.5%) and 6.3 ± 0.6 μL per sample zone (CV 9.2%), respectively, and generated plasma with sub-microliter standard deviations. Here, we sub-sampled the total plasma volume available as a strategy to meter the recovered plasma volume, resulting in three- and four-zone PMCs performing as lead candidate cards.

We simultaneously evaluated ADx 100 cards to compare the metering capabilities of PMCs to a commercial predicate. As ADx 100 cards are manufactured from a single layer of a membrane, plasma flows across these cards with an undefined wicking front, and sample zones must be identified by visual inspection, varying from card to card and patient to patient. When applying 150 μL of blood, we were able to consistently recover four 6 mm punches from each ADx 100 card (Fig. S2). Across the full range of hematocrits, these punches collected 5.1 ± 1.3 μL (CV 25.8%) of plasma (Fig. 2). Though this volume was the smallest amongst the cards, the volume of saturation (*i.e.*, recovered volume per sample area) of the ADx 100 card was 0.18 μL mm^−2^, roughly equivalent to those of the one-, two-, and three-zone PMCs (Fig. S1). The four-zone PMC had a slightly lower volume of saturation, 0.16 μL mm^−2^; however, the precision of volume collected in a four-zone PMC was superior to that of the ADx 100, with a 2.5-fold reduction in overall variation across all samples (9.2% *vs.* 25.8% CV, respectively). When comparing intra-card variation within the three- and four-zone PMCs and ADx 100 cards, the average CV within each card was 6.3% for the PMCs and 22.7% for the ADx 100 cards (Fig. S7). This difference was likely due to chromatographic effects caused by the uncontrolled flow in the ADx 100 cards, which was readily observed in Fig. S2A. The inter-card variation for the ADx 100 is 2-fold higher than for the three- and four-zone PMCs—the CV between the average sample zone volume in each card was 6.8% for the three- and four-zone PMCs and 12.6% for the ADx 100 cards. These data support two key performance criteria that are informed by the design and construction of the PMC: (i) subsampling the total volume with smaller punches, like in the ADx 100 card, did not lead to improved precision. Dividing the plasma sample by patterning, like in the PMC, helped to guide the flow of plasma into sample zones for increased precision regardless of the number of sample zones in the card. (ii) Patterning removed spatial bias in volume collection and led to sample zones that are interchangeable with respect to volume metered.

### Aggregate volume and overall yield of collected plasma

**Fig. 3:**
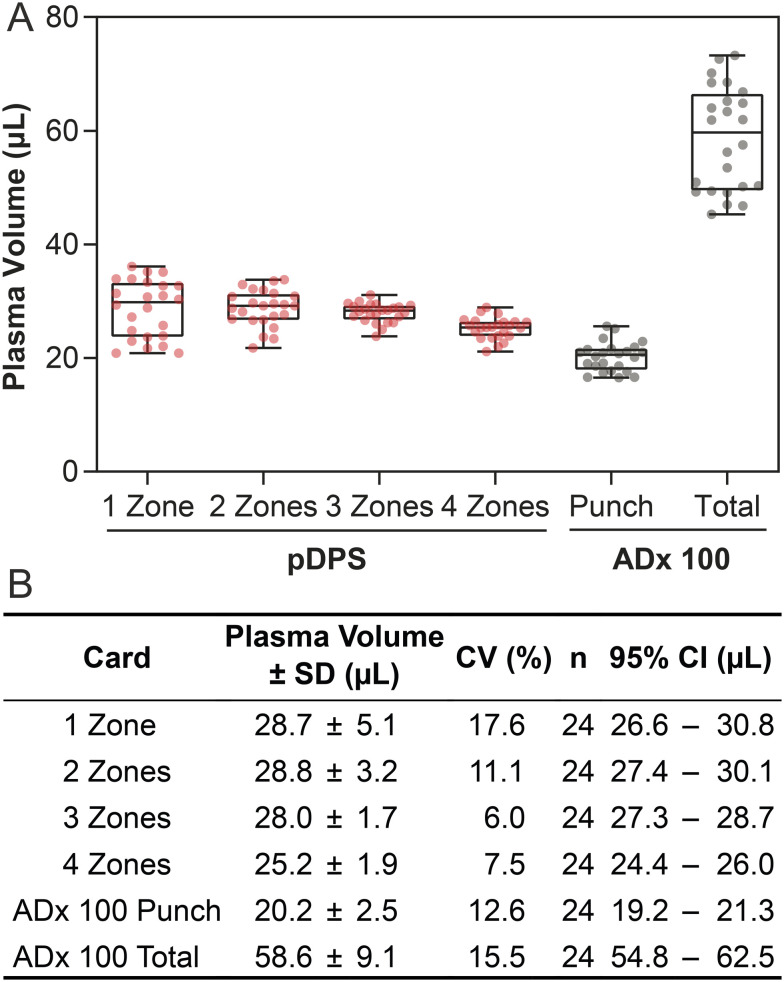
Plasma volumes aggregated from individual sample zones from PMCs (various numbers of zones) and 6 mm punches from ADx 100 cards over the full range of hematocrits (30–55%). (A) Box plot of plasma volumes collected in different card formats and types (PMC in red, ADx 100 in gray). The whiskers represent the minimum and maximum measured volumes. ADx 100 cards generated a larger total volume of plasma compared to PMCs, but the plasma could not be sampled in its entirety from sub-punches; approximately two-thirds of the plasma area was not accessible with 6 mm punches (standard method for recovery of samples from ADx 100 cards). (B) Table of summary statistics from measurements of plasma from aggregated samples, including standard deviation (SD), coefficients of variation (CV), the number of replicate measurements for each card (*n*), and 95% confidence interval (CI). One-, two-, and three-zone PMCs all collected similar volumes, while four-zone PMCs collected ∼3 μL less on average.

We anticipated that assays requiring larger sample volumes (*e.g.*, due to low concentrations of analyte) may need more volume than is contained within a smaller sample zone. We sought to evaluate whether sample zones could be aggregated to generate samples with larger volumes without impacting sampling precision. We aggregated the volumes from the individual sample zones from two-, three-, and four-zone PMCs and compared recovered volumes to those of a one-zone card (Fig. 3). Since we designed the candidate PMCs to standardize the total collection area over all four iterations, we anticipated that the total plasma volumes collected by all PMCs would be similar. The aggregate volumes from the two- and three-zone PMCs were 28.8 ± 3.2 μL (11.1% CV) and 28.0 ± 1.7 μL (6.0% CV), respectively, both of which were within one standard deviation of the average recovered volume from the one-zone card, 28.7 μL. The volume collected from the four-zone card, however, was roughly 3 μL less than the other PMCs, 25.2 ± 1.9 μL (7.5% CV). This result is supported by the approximately 10% reduction in volume of saturation seen in the four-zone cards. We can potentially attribute this difference to the consequences of scaling the card design. While the total sample collection area was identical for all four patterned cards, the four-zone PMC had the most channels, each with an area of 15.1 mm^2^, which then produced more dead volume than the PMCs with fewer sample zones (Fig. S1).

When we aggregated the volumes recovered from four punches of the ADx 100 cards, the total volume was 20.2 ± 2.5 μL (12.6% CV). While all cards had smaller errors in the aggregated samples, the CVs for the ADx 100 cards were still approximately double that of the three- and four-zone PMCs. Moreover, this volume was substantially lower than the total volume of plasma generated by the ADx 100 card. The total sampling volume, as determined by image analysis of the saturated area, was 58.6 ± 9.1 μL (15.5% CV). Two major drawbacks of the ADx 100 cards were the inconsistency in how the separated plasma fills the membrane and the reliance on the user to identify where to retrieve samples from the membrane; we found that we could retrieve only four 6 mm punches from the ADx 100 cards reliably across all six hematocrits. Consequently, while the total plasma volume generated by the ADx 100 card is, on average, around 70% of the available plasma in the sample, the aggregate volumes recovered from four circular punches only represent around 24% of the available plasma in the sample. The operational efficiency of the PMCs consistently outperformed that of the ADx 100, as they yielded around 33% of the available plasma in the one- to three-zone cards and 30% in the four-zone cards (Table S1).

### Hematocrit independence of collected plasma

A significant challenge in metering plasma in microsampling cards is that the amount of plasma available varies widely from patient to patient. In the case of an input of 150 μL, the available plasma from a sample with 30% hematocrit is 105 μL, while a sample with a hematocrit of 55% is 67.5 μL, a 64% decrease in available plasma. To investigate how PMCs could mitigate the challenge associated with the potential for a substantial variance in accessible volume, we characterized plasma recovery as a function of hematocrit (30–55%) at a fixed input volume (150 μL). For one- and two-zone PMCs, we observed two distinct populations of collected volumes: one with samples prepared at 30–45% hematocrit and one with samples prepared at 50–55% hematocrit. One-zone cards operated at 30–45% hematocrit produced plasma volumes on average 3.0 μL above the global average, while the cards at 50–55% hematocrit produced plasma volumes 6.1 μL below the global average (Fig. S8). With two sample zones, this effect was lessened with a 5.2 μL and 2.6 μL difference in aggregated and individual sample volumes between cards operated at 30–45% and 50–55% hematocrit (Fig. S8). However, these differences still represent roughly 20% of the expected volume from these PMCs.

**Fig. 4:**
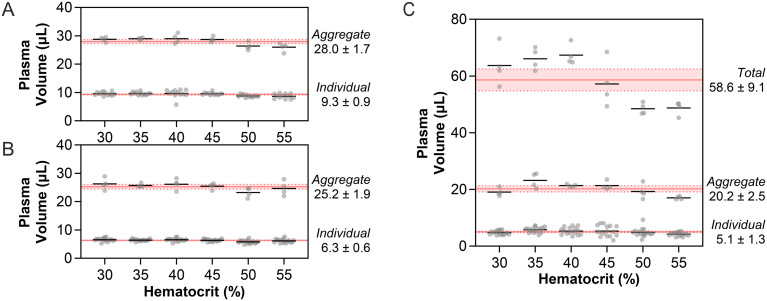
Impact of hematocrit on recovered plasma volumes. Individual and aggregate volumes of (A) three-zone PMCs, (B) four-zone PMCs, and (C) ADx 100 cards. Each card type was evaluated in quadruplicate (*n* = 4) for each of the six levels of hematocrit (30–55%), affording *n* = 24 total cards per type. The shaded region indicates the global mean ± standard deviation for each card type.

Three-zone PMCs produced little difference between 30–45% and 50–55% hematocrit in individual sample zones, 0.9 μL, as well as in aggregate plasma volumes, 2.7 μL, both of which were 9.6% of the overall average volume (Fig. 4A). Four-zone PMCs performed the best across hematocrits, with a 0.5 and 1.9 μL difference for individual and aggregated plasma volumes, 7.8% of the overall average volume (Fig. 4B). These PMCs produced a difference in recovered plasma volume of roughly 5% of the original 37.5 μL plasma difference in total available plasma due to hematocrit, effectively mitigating its influence on recovered plasma volumes. As a result, the three- and four-zone PMCs metered the collected volumes of plasma without a dependence on the hematocrit.

When we analyzed the performance of the ADx 100 cards across the range of hematocrits, there was a notable difference in card performance, in terms of overall collected volumes, which was subsequently reduced when subsampling the collection area. Individual punches from the ADx 100 cards were less affected by differences in hematocrit because they contained relatively low volumes of plasma (*ca.* 5.1 μL), so the disparity in recovered plasma at 30–45% *vs.* 50–55% hematocrit was just 0.8 μL (Fig. 4C). However, the hematocrit effect for these cards was more pronounced when the collected volumes were aggregated, as the difference between these hematocrits became 3.1 μL. When considering the total volume of plasma processed by the card, this difference is 15 μL or 25% of the overall average of the total volume. In both cases, ADx 100 cards spotted at the highest hematocrit (55%) generated the lowest volume (Table S2). In large part, these issues could be explained by a decrease in the available plasma sampling area, which made it challenging to retrieve four full 6 mm punches consistently (Fig. S2). These data suggest that the overall performance of the ADx 100 card was hematocrit-dependent, where sampling was highly influenced by the physiological characteristics of the patient (*i.e.*, hematocrit and available plasma volume), the card geometry (*i.e.*, unique shape of the saturated area), and user operation (*i.e.*, ability to identify and recover suitable punch areas). These performance issues can be exacerbated by low-quality samples containing intracellular interferents, which cause additional issues in the precision and accuracy of dried samples with respect to liquid plasma values.

### Quality of collected plasma

**Fig. 5:**
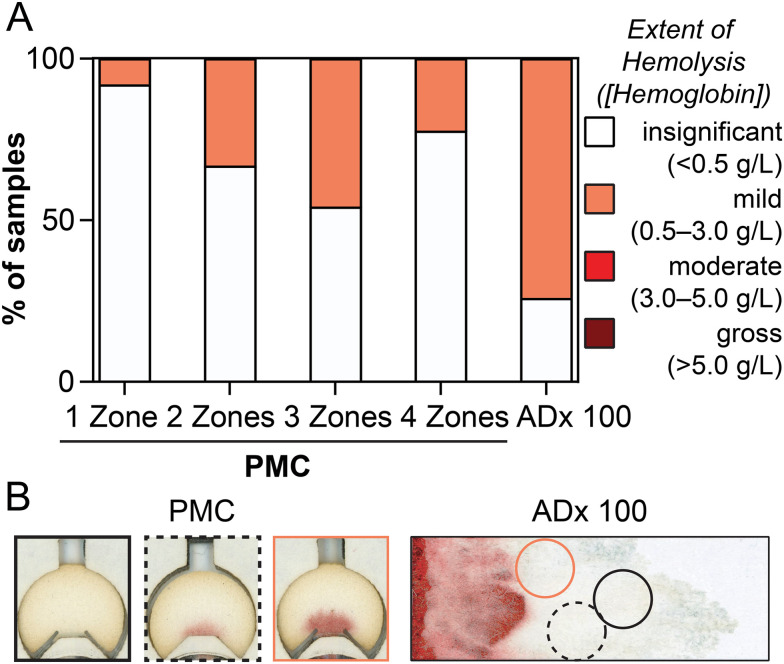
Extent of hemolysis in plasma collected by PMCs and ADx 100 cards. (A) Bar graph depicting the percentage of sample zones or punches with insignificant (<0.5 g L^−1^ hemoglobin; white) and mild (0.5–3.0 g L^−1^ hemoglobin; orange) hemolysis. No samples had evidence of moderate (3.0–5.0 g L^−1^) or gross (>5.0 g L^−1^) hemolysis. (B) Representative scans of PMC and ADx 100 sample zones or punches with hemolysis categorized as insignificant (black outline) or mild (orange outline). The black dashed outline represents a sample at the higher end of insignificant hemoglobin plasma. Each card type was evaluated in quadruplicate (*n* = 4) for each of the six levels of hematocrit (30–55%), affording *n* = 24 total cards per type.

In addition to precision, the quality of the collected dried plasma is also a considerable concern when developing accurate assays. Here, we defined quality as substantially depleted of intraerythrocytic analytes, which we quantified using measurements of hemoglobin to be inclusive of lysis or cellular contaminants. The threshold for acceptable hemolysis is generally defined as containing <0.5 g L^−1^ hemoglobin.^16^ In addition to quantifying hemoglobin eluted from dried plasma sample zones, we also made direct comparisons to a reference sample of liquid plasma from the donor prepared *via* centrifugation as a baseline. We determined that PMCs generate plasma samples in which a majority (>67% per sample zone number) had little to no detectable hemolysis (Fig. 5A). In comparison, 74% of punches from the ADx 100 cards had at least mild levels of hemolysis (0.5–3.0 g L^−1^ hemoglobin). Colorimetric assays like total protein^17^ for unsaturated iron binding capacity^41^ begin to be affected by hemoglobin at concentrations of 0.5 g L^−1^, so samples above this threshold are unfit for these measurements. Sample zones from the PMC where hemolysis was present were characterized by a visual accumulation of hemoglobin (*i.e.*, red band) localized where the patterned channel fed the sample zone. In an ADx 100 card, however, hemolysis could not be quantified with such an obvious indicator, leaving the technician retrieving the sample to judge where best to punch. These observations suggest that visual inspection of PMC sample zones could function as a quality control metric (Fig. 5B). The overall low levels of hemolysis in our PMCs are promising for their intended use in a range of different tests.

## Conclusions

We developed a set of PMCs that can reproducibly collect high-quality dried plasma for downstream analytical applications in clinical laboratories. These cards provided a flexible format for measuring panels of multiple analytes, performing test replicates, confirmation testing, or even biobanking efforts. All PMCs analytically outperformed ADx 100 cards with respect to intra- and inter-card precision, hematocrit-independent metering, and plasma quality. Moreover, the design of the PMC offers additional benefits for both analytical and clinical workflows. PMCs provide fixed sampling locations that minimize variation between experiments. Collecting a sample from a defined position is easier for technicians, since there are no judgment calls on where they should retrieve the sample from the card. This design element reduces the number of decisions that need to be made and mitigates the risk of sampling bias from technician to technician. Multi-zone PMCs also facilitated metered subsampling of the total available plasma sample across all six hematocrits without sacrificing plasma quality. Other plasma collection cards have demonstrated that subsampling plasma by removing the separation media (*i.e.*, membranes) before drying results in closely metered plasma volumes (CV < 5%).^27^ While such cards have slight performance improvements, PMCs offer the benefit of metering autonomously without requiring additional user interventions, such as timing plasma separation or the operational step of removing the separation media and handling wet blood. Reducing the complexity of user steps—or eliminating those steps altogether—is critical to the adoption of microsampling cards by a broad population of untrained users.

PMCs demonstrated acceptable analytical performance despite limitations caused by manual, bench-scale fabrication methods that can lead to slight differences in the construction of the cards (*e.g.*, alignment and placement of materials). Moving away from fabrication techniques that are standard to paper-based microfluidic devices, such as wax-patterning, could potentially reduce the variance seen in these cards even further. We attribute these fabrication errors to the failures we observed (Fig. S3). While we excluded devices that were underfilled from our primary analysis, sample zones that were filled in an otherwise rejected device still collected the desired volume of plasma. For example, filled sample zones in four-zone PMCs recovered similar volumes (5.8 ± 0.8 μL) to the successful cards (6.0 ± 0.6 μL) at the same hematocrits (50–55%) (Fig. S9). These volumes were not found to be statistically different from each other by Welch's unpaired *t*-test (*p* = 0.5). These data indicate that even if one sample zone within the PMC failed, the other sample zones could still be used, which is an additional benefit to using PMCs with multiple zones.

PMCs have the potential to provide metered, dried plasma in a format that is easy to use for both patients and clinical personnel. However, the successful translation of PMCs from research laboratories into real-world environments will require additional efforts in human-centered design.^42^ For example, testing PMCs with fingerstick blood will better match their intended use-case and give users an opportunity to provide feedback. To this end, future studies could use PMCs treated with anticoagulants (*e.g.*, lithium heparin), which have been shown to encourage the flow of blood in paper.^43^ Blood microsampling devices must also produce samples that meet specimen acceptance criteria to support testing.^44^ We have shown that PMCs address this issue by directing the flow of plasma to defined sample zones, which ultimately can limit the number of rejections caused by the presence of an insufficient sample volume (*i.e.*, quantity not sufficient; QNS).^45^ Overall, we anticipate that the opportunity to collect dried samples of metered, pure plasma whose volumes are independent of the physiological differences between patients and whose handling can minimize operator bias will have wide-ranging clinical utility. Specifically, the ability to decentralize sample collection without sacrificing sample quality can support the increased demand for indirect-to-consumer health and wellness testing as well as increase access to participation in clinical trials. These changes can be particularly impactful in resource-poor areas, where remote sampling has the greatest impact on access to testing.

## Conflicts of interest

G. G. M., K. R. B., and C. R. M. are co-inventors on patent applications that describe dried plasma spot cards, including the device presented here. C. R. M. has equity and a leadership role in Health Made Easy.

## Supplementary Material

LC-026-D5LC00844A-s001

## Data Availability

Data for this article are available at Zenodo at https://doi.org/10.5281/zenodo.17057926. Supplementary information (SI): contents include: materials and methods. Card scans and schematics. Calibrations for plasma volume and purity analysis. Detailed breakdowns of card performance. See DOI: https://doi.org/10.1039/d5lc00844a.
