# Supplementary material for: Multi-zone patterning enables hematocrit-independent precision metering of dried plasma for decentralized diagnostics
Source: Lab Chip. 2025 Nov 17;26(2):306–15. doi: 10.1039/d5lc00844a (PMC12687116; doi:10.1039/d5lc00844a)
Supplement: LC-026-D5LC00844A-s001 [file LC-026-D5LC00844A-s001.pdf]

Supplementary Information (SI) for Lab on a Chip.
This journal is © The Royal Society of Chemistry 2025

ELECTRONIC SUPPLEMENTARY INFORMATION

Multi-zone patterning enables hematocrit-independent, precision metering of dried plasma for decentralized diagnostics

Amanda Code, Giorgio Gianini Morbioli, Keith R. Baillargeon, Jack Soloway, Yanran Sun, and Charles R. Mace*

Department of Chemistry, Tufts University, Medford, MA 02155

* corresponding author email: charles.mace@tufts.edu

Pages: 22

Figures: 9

Tables: 2

Contents: Materials and Methods. Card scans and schematics. Calibrations for plasma volume and purity analysis. Detailed breakdowns of card performance.

Materials and Methods

Chemical Reagents and Materials

We purchased Fellowes and Avery self-laminate sheets from Amazon. We purchased ¼” clear acrylic sheets from McMaster-Carr. We purchased Vivid PSM grade GR and Leukosorb B from Pall Corporation. We purchased Flexmount Select DF051521 (permanent adhesive double-faced liner) and Flexmount FLEXmark FLX001172 (release liner) from FLEXcon. We purchased ASTM Type I water from Ricca Chemical Company. We purchased Whatman CF12 from Sigma Aldrich. We purchased ADx 100 100 cards from Advanced Dx (Scottsdale, AZ). We obtained samples of whole blood collected in lithium heparin vacutainers from Research Blood Components (Watertown, MA). We purchased 2,4,6-Tris(2-pyridyl)-s-triazine (TPTZ), mercuric nitrate, ferrous sulfate heptahydrate, sodium sulfate and sulfuric acid from Sigma Aldrich. We purchased a Hemoglobin Colorimetric Assay Kit from Cayman Chemical.

Measuring and Contriving Hematocrit

We measured the initial hematocrit of the whole blood sample using the microhematocrit method to determine packed cell volume. To prepare these samples, we filled 40-mm capillary tubes (n = 6) with 3–5 µL of whole blood and sealed one end with capillary tube wax plates (Globe Scientific). We then processed these tubes via centrifugation at 12000 RPM for 3 minutes using a ZipCombo centrifuge (LW Scientific). Afterward, we acquired images with an 8-bit EPSON Perfection V600 PHOTO scanner with a resolution of 800 DPI. We then analyzed images in ImageJ to measure the ratio of the length that the packed cell volume occupied in the tube to the total sample length to calculate the hematocrit of the sample.

To better assess the performance of plasma metering cards (PMC) over a large population of patients, we contrived the hematocrit of blood samples to comprise a range from 30–55%. Hematocrit can be altered by extracting or adding plasma, based on the initial volume of blood and the ratio of the intended and initial hematocrits (Equation 1).

Equation 1. $$V_{plasma} = V_i \left(1 - \frac{HCT_i}{HCT_f}\right)$$

To adjust the amount of plasma, we processed whole blood samples via centrifugation at 800 g for 10 minutes to separate plasma from cells by sedimentation before extracting or adding plasma from that donor. We confirmed the new hematocrit of each sample by the microhematocrit method outlined above (n = 2).

PMC Construction

We used Adobe Illustrator to design our PMCs, based on designs made previously.[1] PMCs were tiled over sheets such that they can be made in batches of 12. All layers were cut on a laser engraving machine (OMTech Maker 50W CO_2 laser engraver), with settings specific to each material type. To construct our paper collection layer, we first cut the CF12 paper (60mm/min, 30% power) to define plasma sample zones. Next, we patterned the hydrophobic barriers on pre-cut CF12 sheets using a double-sided wax transfer method.[2] We printed the front and back wax-patterns on Avery laminate sheets with Xerox ColorQube 8580 wax printer and aligned them with the pre-cut CF12 with the aid of a custom acrylic alignment jig. We then transferred the patterns from the laminate sheets to the cut CF12 sheet with heating using a Promo Heat CS-15 T-shirt press (60 s at 285 °F), which allowed the melted wax to coat the paper fibers. After cooling, we added laser cut layers of liner (60mm/min, 30% power) and Fellowes laminate (60mm/min, 40% power) to the back of the paper layer aligned with the custom jig. We then assembled the top of the cards by manually adding cut disks of PSM (100mm/min, 20% power) and Leukosorb (60mm/min, 12% power) (Cytiva) with a layer of double-sided adhesive (60mm/min, 30% power) in between each layer. After placing a layer of release liner and Fellowes laminate to the front of the cards, we placed the batch of cards through a VEVOR laminator on the cool setting.

Chloride Assay for Volume Determination

To measure chloride eluted from dried plasma samples, we developed an in-house colorimetric plate assay based on the Fried Method, which has been developed commercially.[3] Briefly, we prepared 1 L of assay reagent by adding 0.3061 g of TPTZ, 0.1645 g of $Hg(NO_3)_2$, 0.1223 g of $FeSO_4$ and 2.8692 g of Na_2SO_4 to a 1L volumetric flask containing Type I water. Before bringing the reagent solution to the final volume, we added 2.67 mL of concentrated sulfuric acid to adjust the pH. The resulting assay reagent should be pale blue in color after pH adjustment.

To recover dried plasma, we added all sample zones and punches into tubes containing Type I water (2.4 mL for one-zone samples and 1.2 mL for all other samples) and let extract on a shaker plate for 1 hour. We also created liquid control samples for all three donor samples by diluting known volumes of plasma into the same volumes of Type I water (2.4 mL for plasma samples above 15 µL; 1.2 mL for all plasma samples below 15 µL). Since the plasma from these three donors was not statistically different (**Figure S4**) we pooled plasma for the dried calibration curves. We made these curves by spotting sample zones with known volumes of plasma pooled from all three donors. We handled these calibration zones identically to their respective sample zone sizes (**Figure S4**). After an hour, we added 50 µL of each eluent sample to 150 µL of assay reagent in a 96-well plate, with two technical replicates for each sample. We incubated plates for 10 minutes before measuring absorbance at 620 nm on a Varioskan LUX plate reader.

Image Analysis of ADx 100 cards

We measured total plasma volume available in the ADx 100 cards via image analysis of the total area of material saturated by plasma (**Figure S5**). We analyzed images in Adobe Photoshop by using the magic selector tool to select the plasma area of each sample and highlight it bright green. After creating this mask, we processed resulting images in ImageJ to find the average pixel area of the bright green area using color thresholding in HSB color space (H: 60–

110 Pass, S: 20–225 Pass, B: 0–225 Pass). In the absence of a blank measurement, the standard deviation of the lowest measurand was used to calculate the limit of quantification.

Hemoglobin Assay

To measure hemoglobin, we used a commercial assay from Cayman Chemical that converts the hemoglobin to hematin which forms a stable complex with Triton X-100.[4] We chose this assay since the manufacturer reports a low limit of detection (0.05 g/L) which we saw as suitable for low concentrations of hemoglobin in plasma. We extracted samples directly into 200 µL of the detector solution in order to mitigate any loss of sample. After mixing for one hour, we used empty SpinX extraction columns to recover the liquid from the paper sample zones via centrifugation at 13,200 g for 2 minutes. We then added 150 µL of the recovered volume into a 96-well plate and measured the average absorbance from 560–590 nm. To control for elution differences, we prepared dried sample controls of clean plasma for each card type by drying 20 µL of plasma in the sample zone. We also constructed hemoglobin calibration curves for the PMC sample zones, since there was non-negligible interference from artifacts of laser cutting in the sample. The ADx 100 cards were not affected by this interference so we only used a liquid calibration for those samples. All calibrations from these samples had sufficient performance to use in our experiments (**Figure S6**).

Outlier removal in chloride measurements

Since we conducted chloride measurements with a homemade assay, we identified and removed outliers that were likely due to inconsistencies in the assay itself. In calibration curves, we identified outliers after performing least squares linear regression and finding standardized residuals from that regression that exceeded 2.5. Anywhere between 2 and 3 for a cutoff has been reported as reasonable[5]; we decided on 2.5 as it gave the best fit. We removed 4 outliers out of 240 measurements for our dried calibrations and 8 outliers out of 290 measurements for our liquid

measurements. For our experimental data, we used iterative Grubb’s test to remove outliers in chloride data prior to averaging the two technical replicates by comparing sample measurements from replicate devices. To prevent removing any measurement that was an outlier due to device performance, we only removed one chloride measurement from any sample. In this data set, we removed 15 outliers leading to those samples having only one chloride replicate. Of the 336 samples measured only two were flagged as outliers in both measurements by Grubb’s test, and therefore one measurement was kept in the set.

Figure S1. Schematics and images of plasma metering card (PMC) designs. (A) Geometry of PMC sample zones, which scale as the number of zones increases from one to four. (B) Diagram of wax-patterned designs and (C) scans of resulting layers after the wax-melting step. For clarity, we include shared features (e.g., channel dimensions) for only the one-zone card. ADx 100 punches were 6 mm in diameter with an area of 28.3 mm^2.

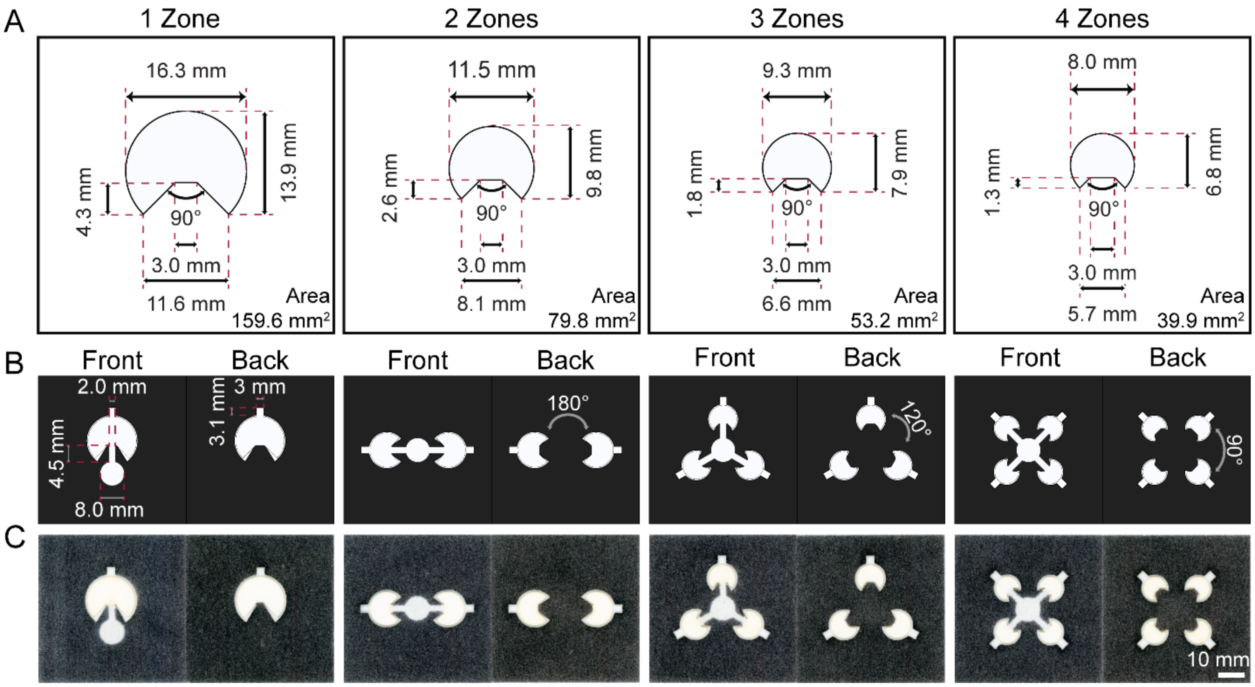

Figure S2. Scans of all plasma separation cards used in this manuscript. (A) ADx 100 cards. (B) One-zone PMCs. (C) Two-zone PMCs. (D) Three-zone PMCs. (E) Four-zone PMCs. Cards for volume and hemolysis studies were processed and analyzed by the chloride and hemoglobin assays, respectively, and were performed in quadruplicate (replicates 1–4). Punches obtained from ADx 100 cards are marked by circles with dashed outlines, while the pattern in PMCs denotes the location of sample zones. Scans for some devices were adjusted in Adobe Photoshop to account for lighting differences.

A

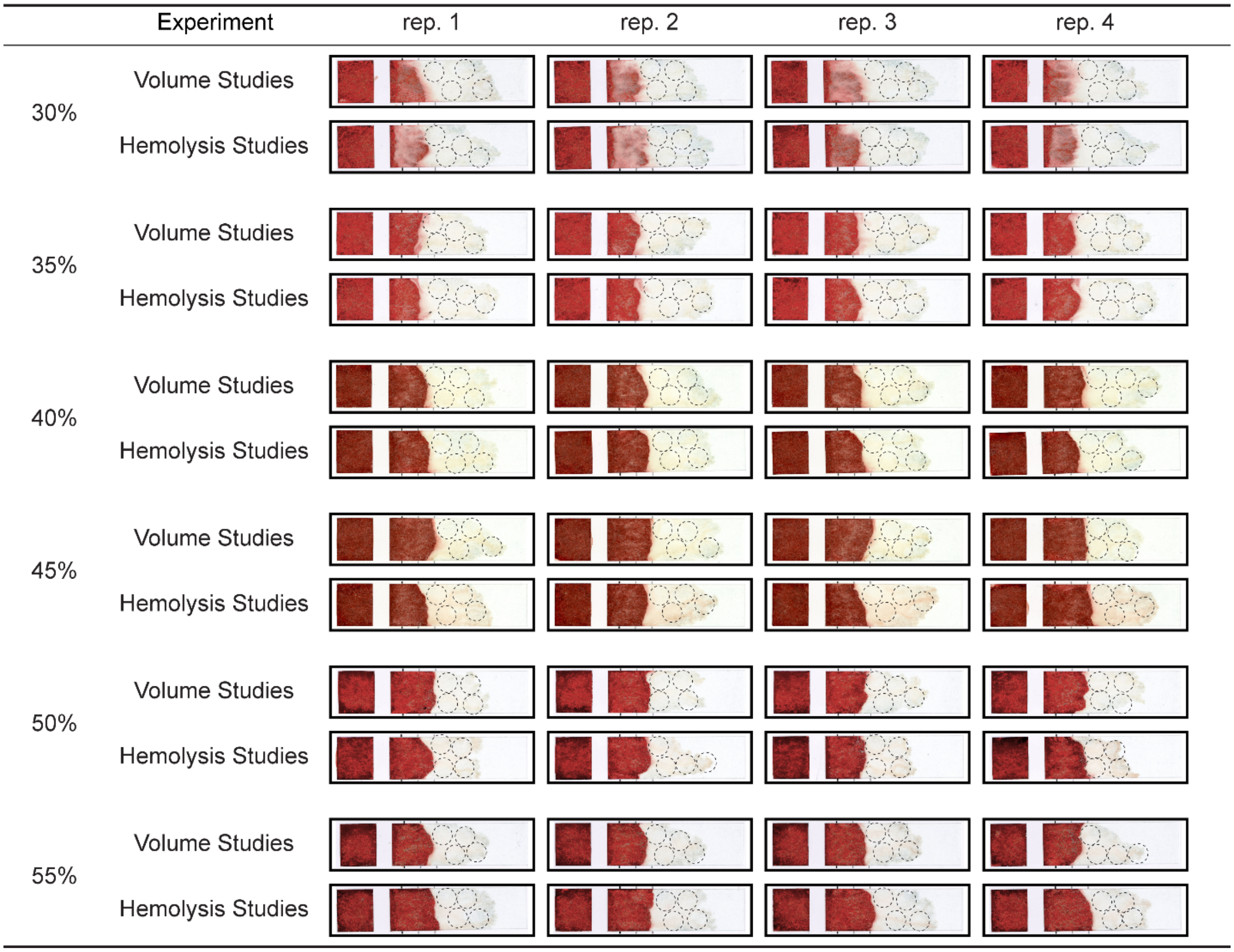

B

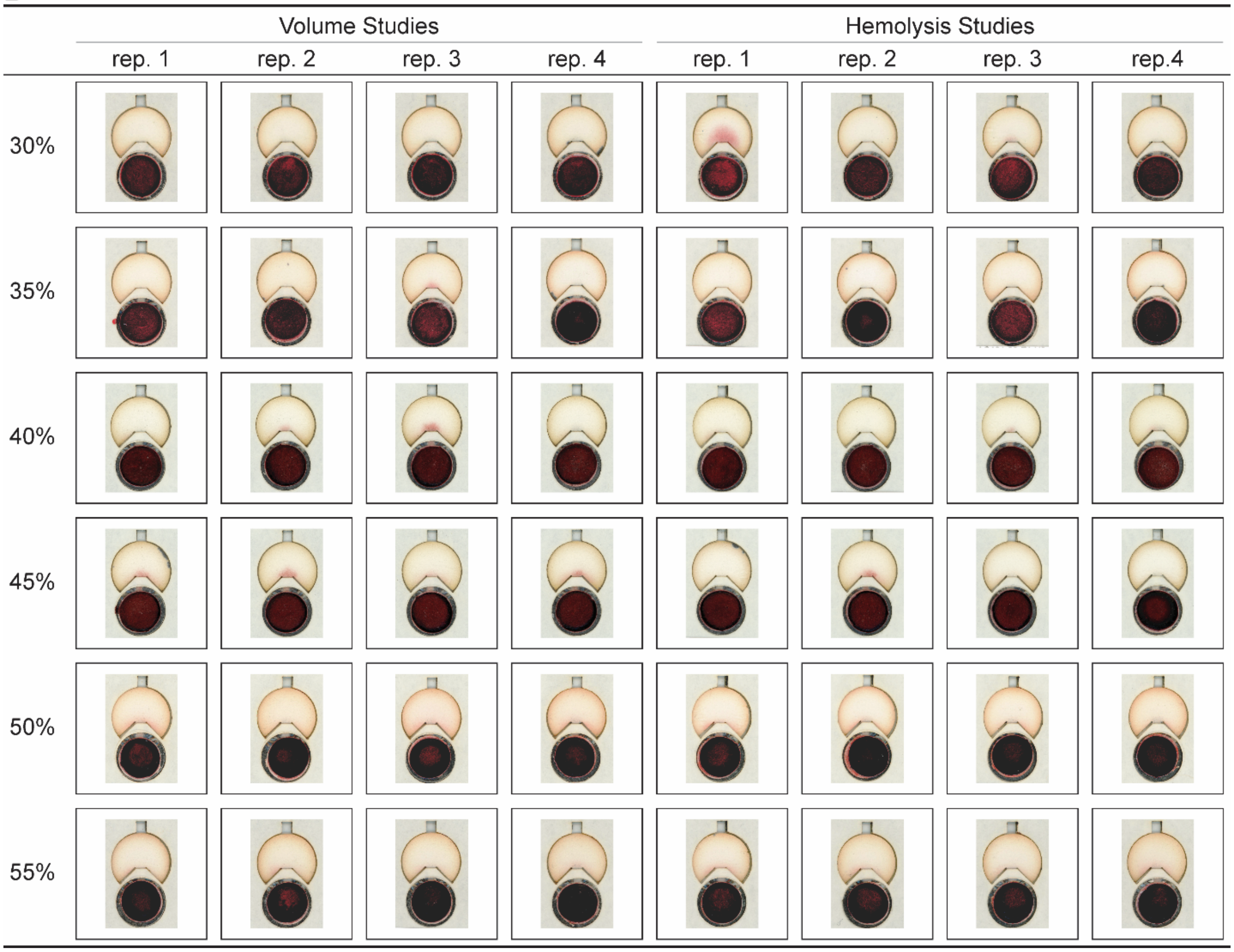

Volume Studies
Hemolysis Studies
rep. 1
rep. 2
rep. 3
rep. 4
rep. 1
rep. 2
rep. 3
rep.4
30%
35%
40%
45%
50%
55%

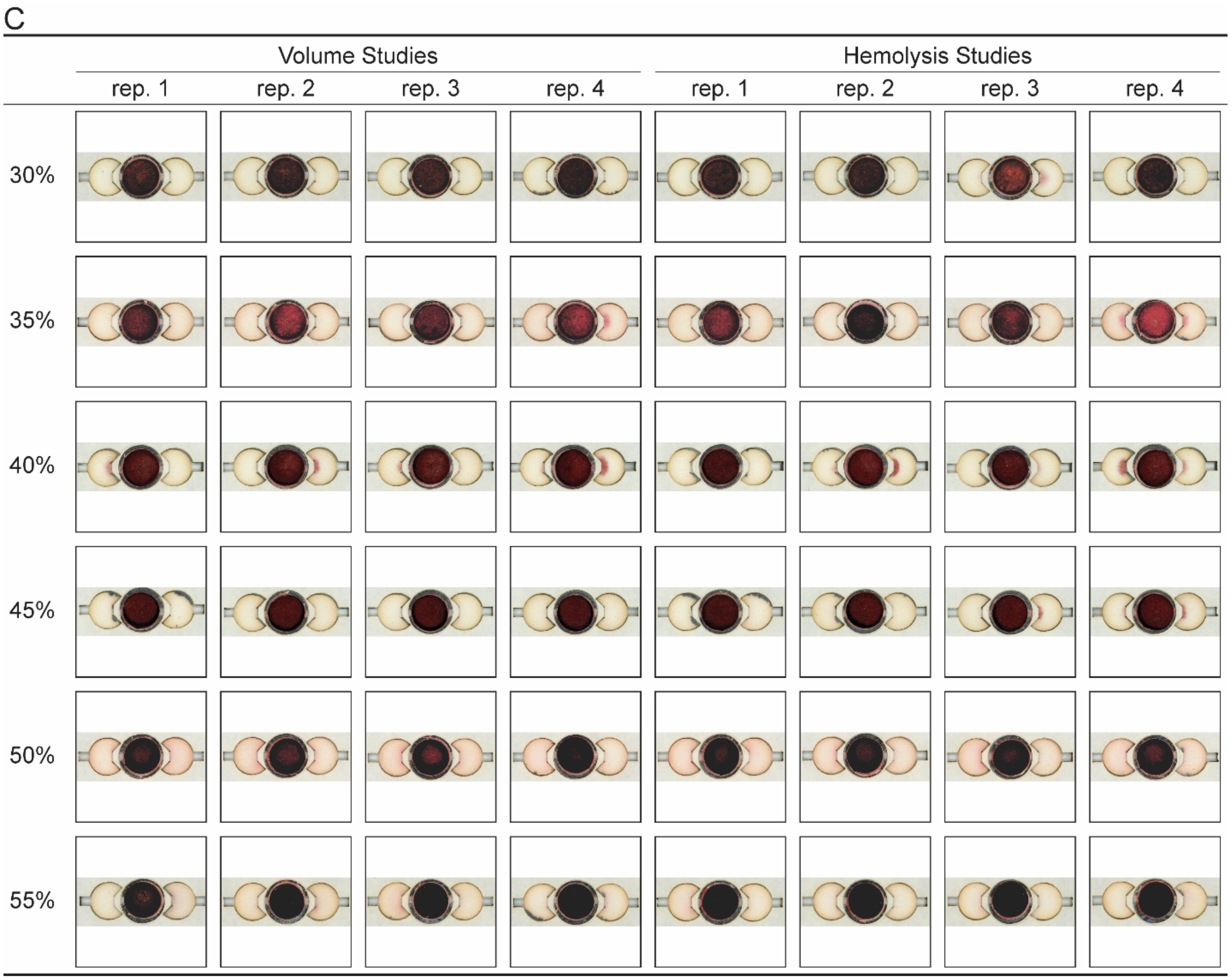
C
Volume Studies
Hemolysis Studies
rep. 1
rep. 2
rep. 3
rep. 4
rep. 1
rep. 2
rep. 3
rep. 4
30%
35%
40%
45%
50%
55%

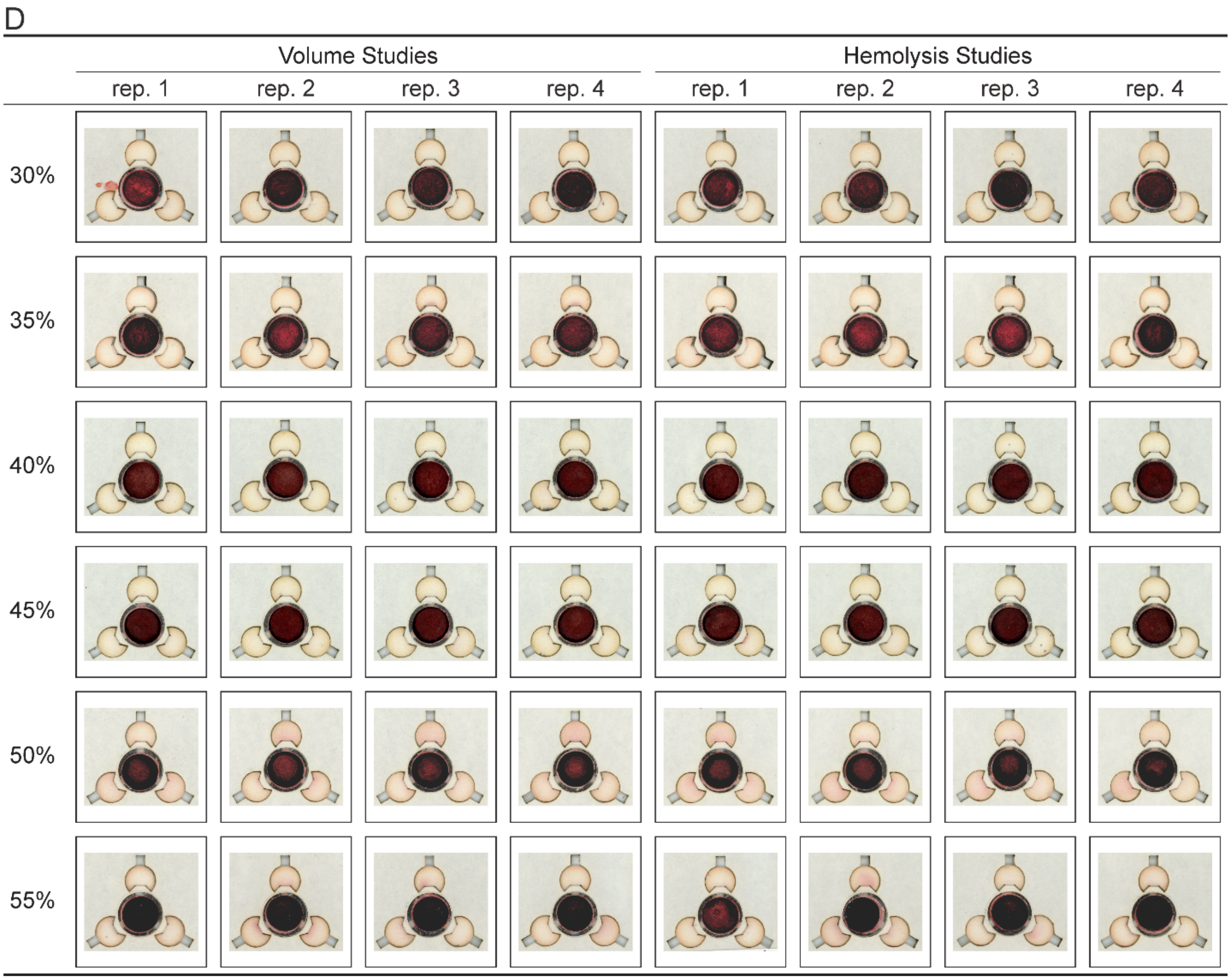
D
Volume Studies
Hemolysis Studies
rep. 1
rep. 2
rep. 3
rep. 4
rep. 1
rep. 2
rep. 3
rep. 4
30%
35%
40%
45%
50%
55%

E

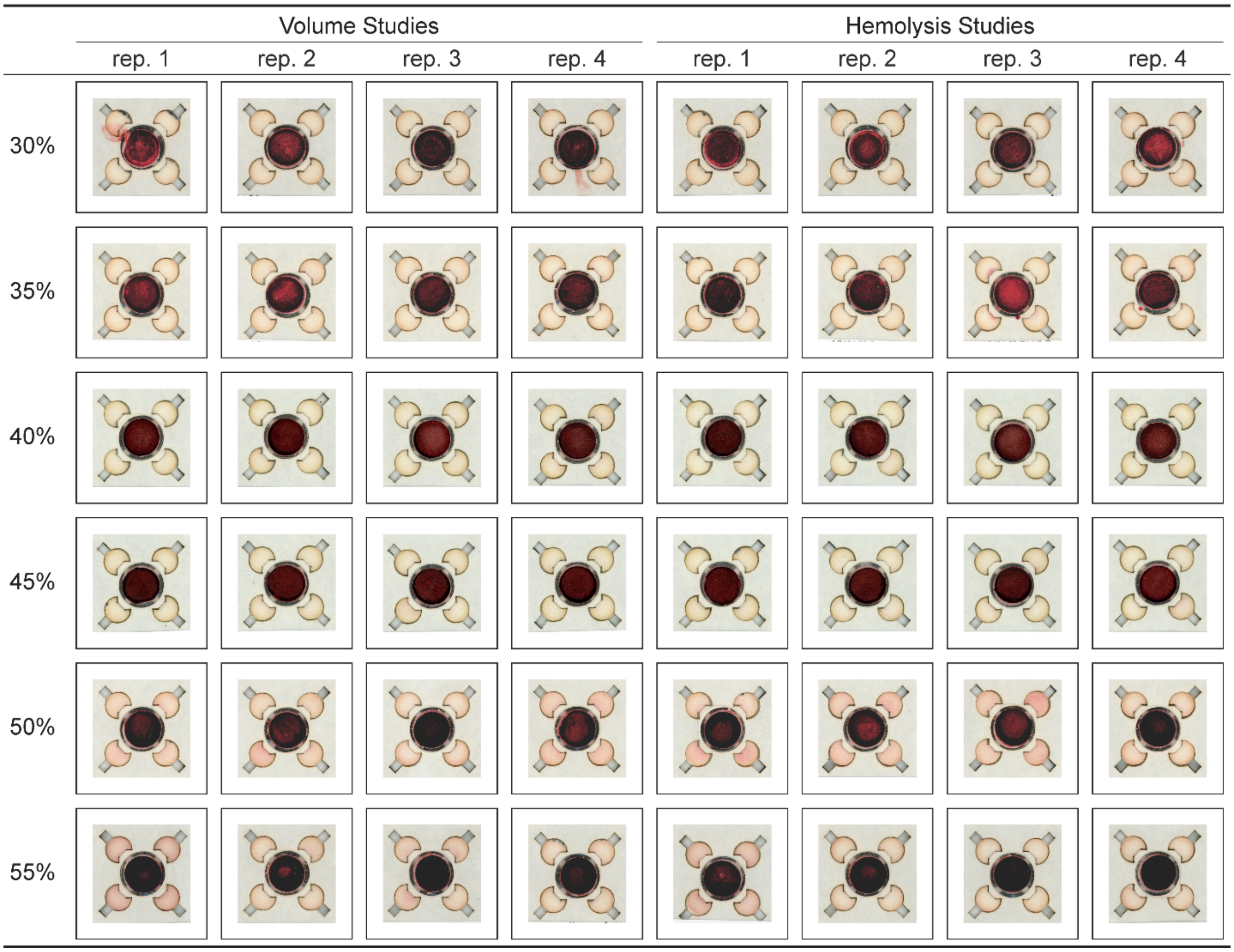
Volume Studies
Hemolysis Studies
rep. 1
rep. 2
rep. 3
rep. 4
rep. 1
rep. 2
rep. 3
rep. 4
30%
35%
40%
45%
50%
55%

Figure S3. Failure rates of different PMC configurations. (A) Rates quantified by hematocrit and categorized by failure mode. (B) Representative scans of cards that visualize successful filling and failures. Results from successful cards were included in the main study while cards with underfilled sample zones or that had no flow were excluded. Errors in card function are likely due to mistakes in bench-scale manufacturing, such as lack of connection between separation media and the paper channels beneath.

A

Card	Hct (%)	Successful	Underfilled	No Flow	Total	% Failed
1 Zones	30	8	0	0	8	0
	35	8	0	0	8	0
	40	8	0	0	8	0
	45	8	0	0	8	0
	50	8	0	0	8	0
	55	8	0	0	8	0
	Sub total	48	0	0	48	0
2 Zones	30	8	0	1	9	11
	35	8	1	2	11	27
	40	8	0	0	8	0
	45	8	0	0	8	0
	50	8	0	0	8	0
	55	8	0	1	9	11
	Sub total	48	1	4	53	9
3 Zones	30	8	0	0	8	0
	35	8	0	0	8	0
	40	8	0	0	8	0
	45	8	0	0	8	0
	50	8	0	0	8	0
	55	8	2	1	11	27
	Sub total	48	2	1	51	6
4 Zones	30	9	0	5	14	36
	35	8	0	0	8	0
	40	8	0	0	8	0
	45	8	0	0	8	0
	50	8	0	5	13	38
	55	7	6	0	13	46
	Sub total	48	6	10	64	25
Total		192	9	15	216	11

B

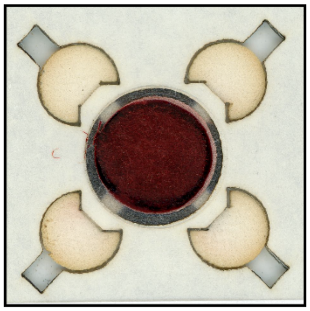

Successful Card

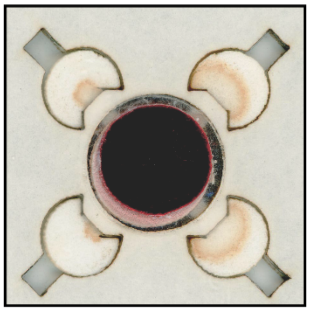

Underfilled Card

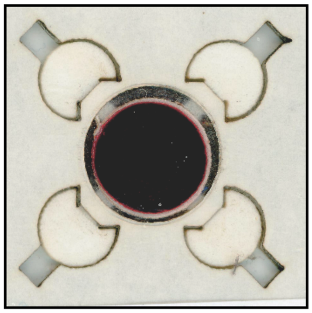

No Flow Card

Figure S4. Calibration curves for volume measurements from (A) liquid plasma (n = 4) and dried samples (n = 6) for (B) one-zone, (C) two-zone, (D) three-zone, and (E) four-zone PMC and (F) ADx 100 cards (H). (G) All plasma volume calibrations together. Calibration curve for chloride measurements and (I) chloride measurements from individual donors, which were not statistically different by one-way ANOVA (p = 0.3). Errors bars represent the standard deviation.

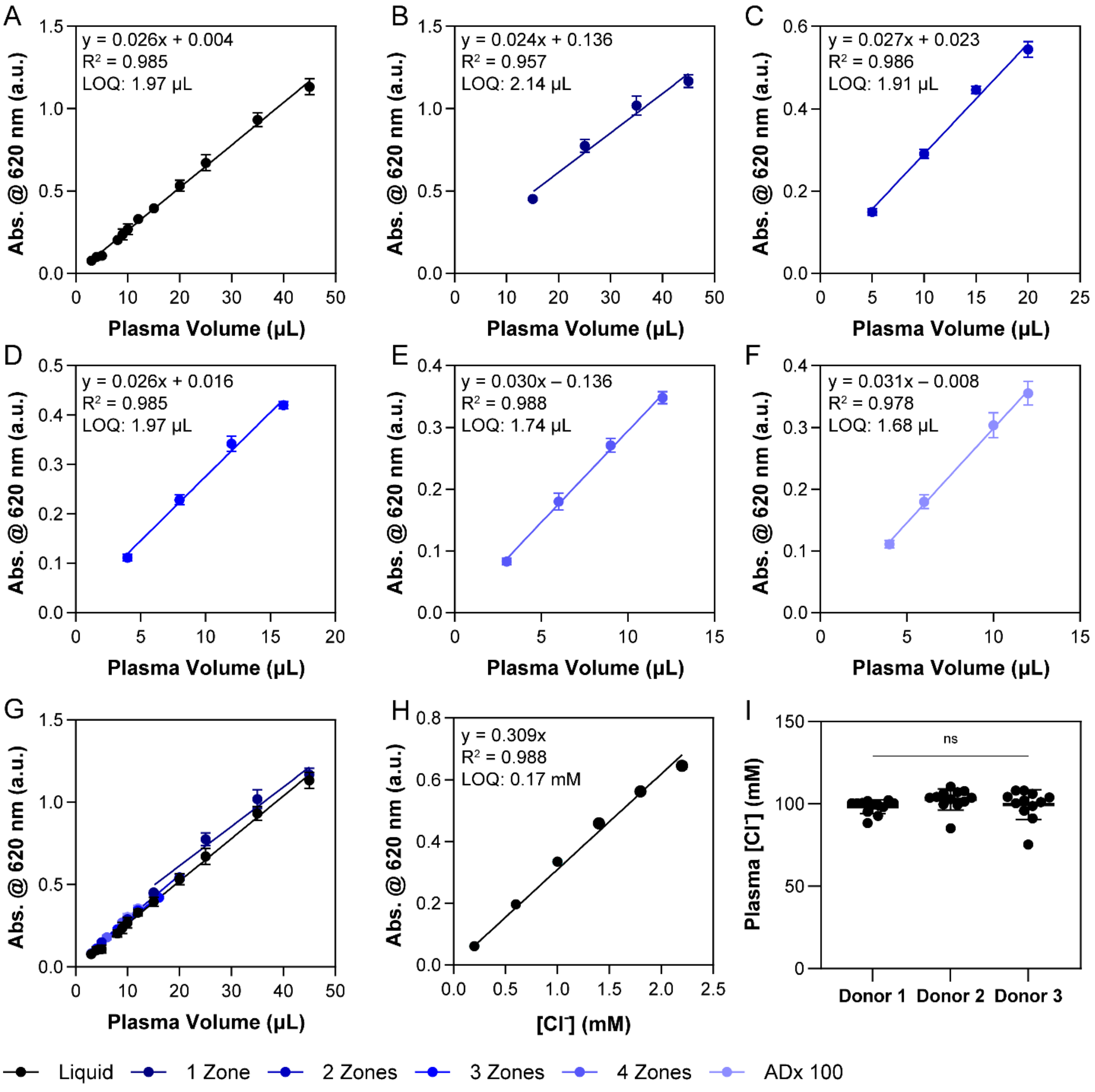

Figure S5. Analysis of total plasma volumes produced by ADx 100 cards from an input of 150 μL of whole blood. (A) Scans of cards depicting the determination of plasma area using masking conducted in Adobe Photoshop. (B) Calibration curves relating pixel area measured in ImageJ as a function of plasma volume (n = 3). Errors bars represent the standard deviation.

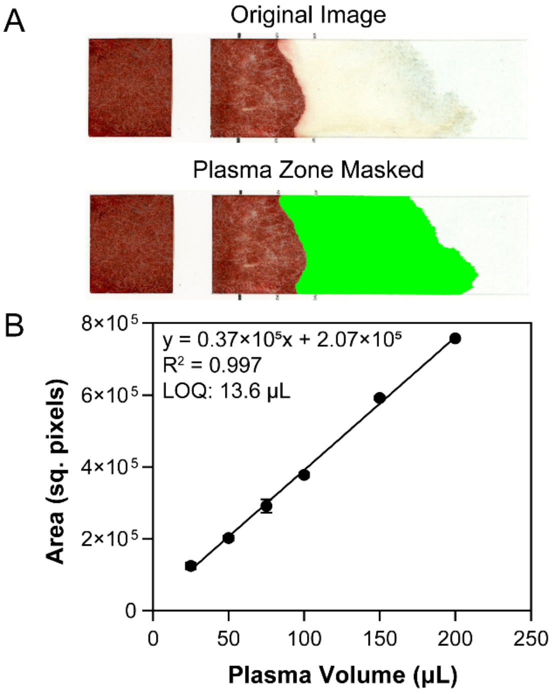

Figure S6. Calibration curves for hemoglobin standards from (A) liquid plasma (n = 4) and dried samples (n = 4) for (B) one-zone, (C) two-zone, (D) three-zone, and (E) four-zone PMC and (F) All plasma volume calibrations together. ADx 100 spots did not interfere with the assay so liquid calibration was found to be acceptable for these samples.

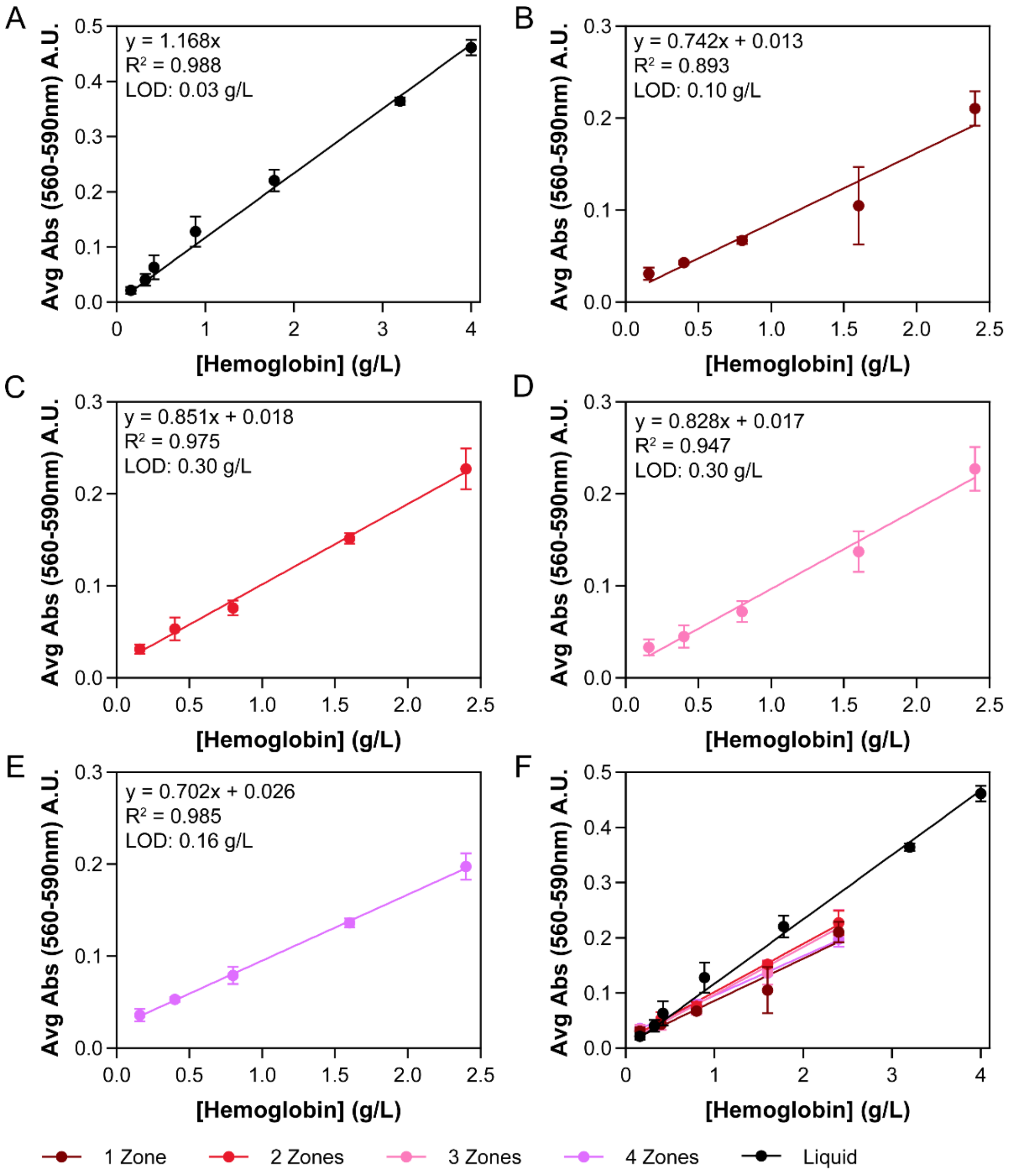

Figure S7. Recovered plasma volumes and intra-card coefficients of variation for different card types as a function of hematocrit. All individual measurements are shown with gray markers with the average of those measurements depicted by a black bar. (A) three-zone PMC (6.7% CV), (B) four-zone PMC (5.9% CV), and (C) ADx 100 cards (22.7% CV). Shaded pink region represents mean with 95% confidence interval across all devices.

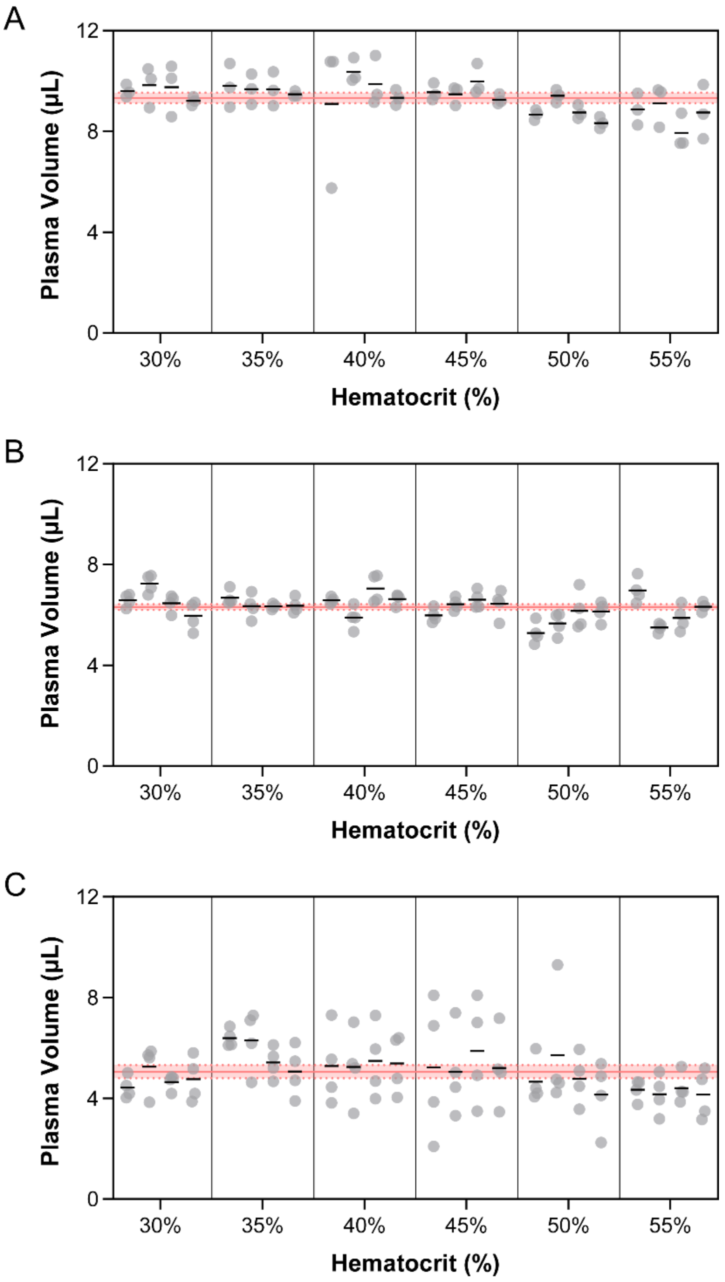

Table S1. Total yield of plasma per card type across all hematocrits.

Card	Yield of Total Plasma (%)						
	30%	*35%*	*40%*	*45%*	*50%*	*55%*	*All*
1 Zone	29.7	33.2	33.4	40.2	31.1	32.5	33.4
2 Zones	28.4	31.3	36.2	35.4	34.3	37.0	33.8
3 Zones	27.4	29.7	32.2	34.8	35.2	38.5	33.0
4 Zones	25.0	26.4	29.1	30.8	31.0	36.5	29.8
ADx 100 Punch	18.2	23.8	23.8	25.9	25.7	25.3	23.8
ADx 100 Total	60.7	67.8	74.9	69.4	64.7	72.2	68.3

Figure S8. Volumes of plasma recovered from (A) one-zone PMCs and (B) two-zone PMCs. By definition, volumes cannot be aggregated from one-zone cards. Four cards were evaluated over the range of hematocrits (30–55%), for a total of 24 cards per type of PMC. All individual measurements are shown with gray markers with the average of those measurements depicted by a black bar. Shaded pink region represents mean with 95% confidence interval across all devices.

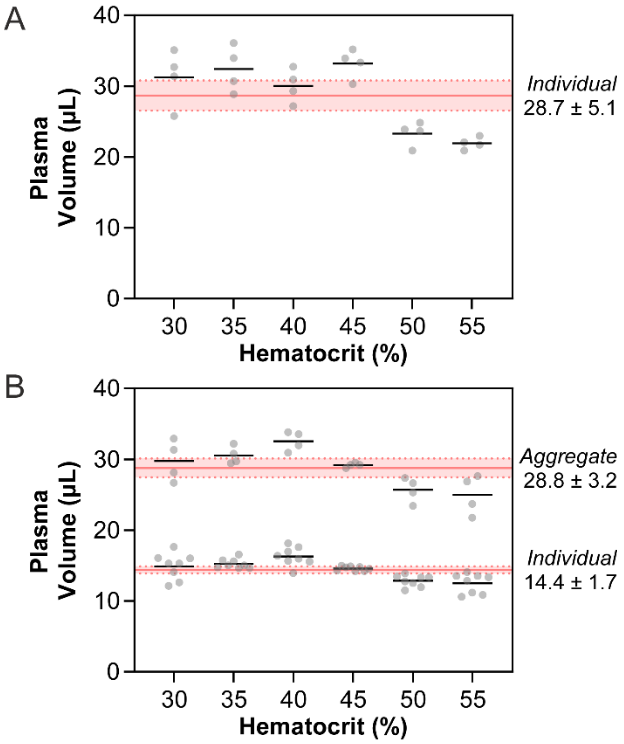

Table S2. Table of summary statistics of all cards assessed the main study, broken out by hematocrit, including standard deviation (SD), coefficients of variation (CV), number of replicate measurements for each card (n), and 95% confidence interval (CI).

Card	Hct (%)	*Individual*				*Aggregate*			
		**Plasma Volume ± SD (μL)**	**CV (%)**	**n**	**95% CI (μL)**	**Plasma Volume ± SD (μL)**	**CV (%)**	**n**	**95% CI (μL)**
1 Zone	30	31.2 ± 4.0	12.7	4	24.9 – 37.5	31.2 ± 4.0	12.7	4	24.9 – 37.5
	35	32.4 ± 3.2	10.0	4	27.3 – 37.6	32.4 ± 3.2	10.0	4	27.3 – 37.6
	40	30.0 ± 2.4	7.9	4	26.3 – 33.8	30.0 ± 2.4	7.9	4	26.3 – 33.8
	45	33.2 ± 2.1	6.2	4	29.9 – 36.5	33.2 ± 2.1	6.2	4	29.9 – 36.5
	50	23.3 ± 1.7	7.3	4	20.6 – 26.0	23.3 ± 1.7	7.3	4	20.6 – 26.0
	55	21.9 ± 0.9	3.9	4	20.5 – 23.3	21.9 ± 0.9	3.9	4	20.5 – 23.3
2 Zones	30	14.9 ± 1.9	12.5	8	13.3 – 16.4	29.8 ± 2.9	9.6	4	25.2 – 34.3
	35	15.3 ± 0.7	4.3	8	14.7 – 15.8	30.5 ± 1.2	4.1	4	28.6 – 32.5
	40	16.3 ± 1.3	8.1	8	15.2 – 17.4	32.6 ± 1.4	4.2	4	30.4 – 34.8
	45	14.6 ± 0.3	2.1	8	14.3 – 14.8	29.2 ± 0.3	1.1	4	28.7 – 29.7
	50	12.8 ± 0.8	6.4	8	12.2 – 13.5	25.7 ± 1.7	6.7	4	22.9 – 28.4
	55	12.5 ± 1.4	11.2	8	11.3 – 13.7	25.0 ± 2.7	10.9	4	20.6 – 29.3
3 Zones	30	9.6 ± 0.6	6.5	12	9.2 – 10.0	28.8 ± 0.8	2.9	4	27.5 – 30.1
	35	9.7 ± 0.6	5.7	12	9.3 – 10.0	29.0 ± 0.4	1.4	4	28.3 – 29.6
	40	9.7 ± 1.4	14.8	12	8.8 – 10.6	29.0 ± 1.7	5.9	4	26.3 – 31.7
	45	9.6 ± 0.4	4.7	12	9.3 – 9.9	28.7 ± 0.9	3.2	4	27.2 – 30.2
	50	8.8 ± 0.5	5.2	12	8.5 – 9.1	26.4 ± 1.4	5.1	4	24.2 – 28.5
	55	8.7 ± 0.8	9.7	12	8.1 – 9.2	26.0 ± 1.5	5.9	4	23.6 – 28.5
4 Zones	30	6.6 ± 0.6	9.0	16	6.2 – 6.9	26.2 ± 2.1	7.9	4	22.9 – 29.6
	35	6.4 ± 0.3	5.0	16	6.3 – 6.6	25.7 ± 0.7	2.6	4	24.7 – 26.8
	40	6.5 ± 0.5	8.4	16	6.2 – 6.8	26.2 ± 1.9	7.4	4	23.1 – 29.2
	45	6.4 ± 0.4	6.5	16	6.1 – 6.6	25.4 ± 1.1	4.2	4	23.7 – 27.1
	50	5.8 ± 0.6	10.4	16	5.5 – 6.1	23.2 ± 1.7	7.2	4	20.6 – 25.9
	55	6.2 ± 0.7	10.7	16	5.8 – 6.5	24.7 ± 2.5	10.3	4	20.6 – 28.7
ADx 100	30	4.8 ± 0.7	14.7	16	4.4 – 5.1	19.1 ± 1.4	7.3	4	16.9 – 21.3
	35	5.8 ± 1.0	16.7	16	5.3 – 6.3	23.2 ± 2.6	11.3	4	19.0 – 27.3
	40	5.3 ± 1.3	23.7	16	4.7 – 6.0	21.4 ± 0.4	2.0	4	20.7 – 22.1
	45	5.3 ± 1.9	35.1	16	4.3 – 6.3	21.4 ± 1.5	6.9	4	19.0 – 23.7
	50	4.8 ± 1.5	30.9	16	4.0 – 5.6	19.3 ± 2.6	13.6	4	15.1 – 23.5
	55	4.3 ± 0.7	15.5	16	3.9 – 4.6	17.1 ± 0.5	3.1	4	16.2 – 17.9
						Total			
	30					63.7 ± 7.1	11.1	4	52.5 – 74.9
	35					66.1 ± 3.8	5.8	4	60.0 – 72.2
	40					67.4 ± 3.6	5.4	4	61.7 – 73.1
	45					57.2 ± 8.2	14.4	4	44.1 – 70.3
	50					48.5 ± 2.0	4.1	4	45.4 – 51.6
	55					48.7 ± 2.3	4.8	4	45.0 – 52.4

Figure S9. Assessment of filled sample zones in failed cards. (A) Average recovered volume from filled sample zones in four-zone PMCs that were either completely successful or considered a failure (unsuccessful). All individual measurements are shown with gray markers with the average of those measurements depicted by a black bar. (B) Table of summary statistics from measurements of recovered dried plasma including standard deviation (SD), coefficients of variation (CV), number of replicate measurements for each card (n), and 95% confidence interval (CI). All PMCs in this analysis were conducted with samples of whole blood at 50 or 55% hematocrit because these conditions had PMCs that failed by underfilling. Sample zones that were underfilled were not included in these analyses. These volumes were not statistically different by Welch's unpaired t-test ($p = 0.5$).

A

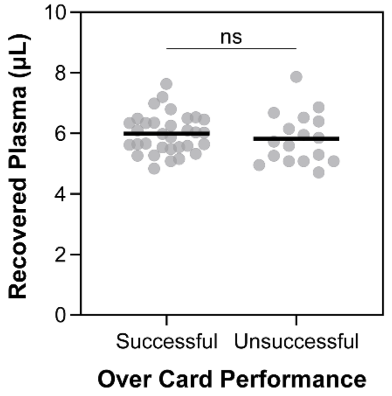

B

Card Performance	Plasma Volume ± SD (µL)	CV (%)	n	95% CI (µL)
Successful	6.0 ± 0.6	10.8	32	5.8 – 6.2
Unsuccessful	5.8 ± 0.8	14.4	17	5.4 – 6.3

References

1. G. G. Morbioli, K. R. Baillargeon, M. N. Kalimashe, V. Kana, H. Zwane, C. van der Walt, A. J. Tierney, A. C. Mora, M. Goosen, R. Jagaroo, J. C. Brooks, E. Cutler, G. Hunt, M. R. Jordan, A. Tang and C. R. Mace, Clinical evaluation of patterned dried plasma spot cards to support quantification of HIV viral load and reflexive genotyping, *Proc. Natl. Acad. Sci. U. S. A.*, 2025, **122**, e2419160122.

2. X. Li, and X. Liu, Fabrication of three-dimensional microfluidic channels in a single layer of cellulose paper. *Microfluid Nanofluid*, 2014, **16**, 819–827.

3. T. W. Stephens, inventors, American Monitor Corporation, assignee. *Assay Method and Reagent for the Determination of* Chloride. International Patent WO 83/02670. 1983-08-04.

4. R. Zander, W. Lang, and H. Wolf, Alkaline haematin D-575, a new tool for the determination of haemoglobin as an alternative to the cyanhaemiglobin method. I. description of the method, *Clin. Chim. Acta*, 1984, **136**, 83–93.

5. M. Ortiz, L. Sarabia, and A. Herrero, Robust regression techniques: A useful alternative for the detection of outlier data in chemical analysis, *Talanta*, **70**, 499–512.
